# Guidelines for diagnostic next-generation sequencing

**DOI:** 10.1038/ejhg.2015.226

**Published:** 2015-10-28

**Authors:** Gert Matthijs, Erika Souche, Mariëlle Alders, Anniek Corveleyn, Sebastian Eck, Ilse Feenstra, Valérie Race, Erik Sistermans, Marc Sturm, Marjan Weiss, Helger Yntema, Egbert Bakker, Hans Scheffer, Peter Bauer

**Affiliations:** 1 Center for Human Genetics, KU Leuven, Gasthuisberg, Laboratory for Molecular Diagnosis, Leuven, Belgium; 2 Department of Clinical Genetics, Academic Medical Centre (AMC), University of Amsterdam, Amsterdam, The Netherlands; 3 Center for Human Genetics and Laboratory Medicine, Martinsried, Germany; 4 Department of Human Genetics, Radboud University Medical Center, Nijmegen, The Netherlands; 5 Department of Clinical Genetics, VU University Medical Center, Amsterdam, The Netherlands; 6 University Hospital of Tübingen, Institute of Medical Genetics and Applied Genomics, Tübingen, Germany; 7 Department of Clinical Genetics, Leiden University Medical Center, Leiden, The Netherlands

## Abstract

We present, on behalf of EuroGentest and the European Society of Human Genetics, guidelines for the evaluation and validation of next-generation sequencing (NGS) applications for the diagnosis of genetic disorders. The work was performed by a group of laboratory geneticists and bioinformaticians, and discussed with clinical geneticists, industry and patients' representatives, and other stakeholders in the field of human genetics. The statements that were written during the elaboration of the guidelines are presented here. The background document and full guidelines are available as supplementary material. They include many examples to assist the laboratories in the implementation of NGS and accreditation of this service. The work and ideas presented by others in guidelines that have emerged elsewhere in the course of the past few years were also considered and are acknowledged in the full text. Interestingly, a few new insights that have not been cited before have emerged during the preparation of the guidelines. The most important new feature is the presentation of a ‘rating system' for NGS-based diagnostic tests. The guidelines and statements have been applauded by the genetic diagnostic community, and thus seem to be valuable for the harmonization and quality assurance of NGS diagnostics in Europe.

Next-generation sequencing (NGS) allows for the fast generation of thousands to millions of base pairs of DNA sequence of an individual patient. The relatively fast emergence and the great success of these technologies in research herald a new era in genetic diagnostics. However, the new technologies bring challenges, both at the technical level and in terms of data management, as well as for the interpretation of the results and for counseling. We believe that all these aspects warrant consideration of what the precise role of NGS in diagnostics will be, today and tomorrow. Before even embarking on acquisition of machines and skills for performing NGS in diagnostics, many issues have to be dealt with. It is in this context that we propose the guidelines. These guidelines mostly deal with NGS testing in the context of rare and mostly monogenic diseases. They mainly focus on the targeted analysis of gene panels, either through specific capture assays, or by extracting data from whole-exome sequencing. In principle, whole-genome sequencing may – and shortly will – also be used to extract similar information. In that case, the guidelines would still apply, but because whole-genome sequencing would also allow detecting other molecular features of disease, they would have to be extended accordingly.

The different aspects of NGS and diagnostics were discussed during three workshops. The first took place in Leuven, 25–26 February 2013. The preliminary views were presented during the EuroGentest Scientific Meeting in Prague, 7–8 March 2013. The second was an editorial workshop in Leuven, 1–2 October 2013, where the different people involved in writing the document came together to discuss the layout of the document and prepare the first draft. The first draft was finalized prior to the third meeting in Nijmegen, 21–22 November, 2013. To the latter meeting, a larger group of stakeholders was invited. They were invited to comment on the draft, and on the statements presented therein. The comments were included in a new version, which was circulated among the editorial group, prior to publication on the EuroGentest and European Society of Human Genetics websites. Eventually, the document was presented to the Board of the European Society of Human Genetics, for endorsement. Endorsement was formally obtained on 1 July 2015.

The statements that emerged during the writing of the guidelines are briefly presented in this paper. They are more extensively explained in the full version of the guidelines, available as supplementary material. The supplementary material also includes definitions, general recommendations and importantly, a number of practical examples and templates.

## State of the art

The available NGS platforms are not stable yet in a sense that the technology and applications change constantly and rapidly. However, this should not prevent the implementation of NGS technology in diagnostics as NGS offers a potential overall benefit for the patient. The one thing that should prevent people from prematurely offering NGS diagnostics is poor quality. Insufficiently validated tests do present a threat to patients, and their use in a clinical diagnostic setting is unacceptable.

**STATEMENT 01: NGS should not be transferred to clinical practice without an acceptable validation of the tests according to the emerging guidelines.**

Whether the aim of a diagnostic test is to exclude or confirm a diagnosis has to be defined beforehand as the distinction is significant. The distinction mainly depends on the completeness of the test and warrants not only different settings but more importantly a different view on diagnostics.

**STATEMENT 02: The laboratory has to make clear whether the test that is being offered may be used to exclude a diagnosis, or to confirm a diagnosis.**

## Diagnostic/clinical utility

The benefit of implementing NGS in diagnostics is the introduction of testing many genes at once in a relatively short time and at relatively low costs, and thereby yielding more molecular diagnoses.

The limitations of NGS are dependent on the platform and on the enrichment methods (if any) and have to be considered as they will influence the choice of enrichment method and sequencing platform and determine which additional tests (if any) will be necessary to deliver high-quality diagnostics.

**STATEMENT 03: The aim and the utility of the test or assay should be discussed at the beginning of the validation and a summary should be included in the validation report.**

The ‘diagnostic yield' is defined as the chance that a disease-causing variant is identified and molecular diagnosis can be made. The value is calculated per patient cohort. It establishes the performance of NGS primarily from a clinical point of view and may be a good indicator of the efficiency of the test (beyond its analytical aspects) and of its clinical utility.

**STATEMENT 04: When a laboratory is considering introducing NGS in diagnostics, it first has to consider the diagnostic yield.**

In practice, diagnostic laboratories will preferably offer gene panels. The conditions for including a gene into a panel have to be defined when developing a diagnostic test. Ideally, this is an issue that should be dealt with at the community level, in a multidisciplinary way. The aim is to compile the list of genes that should be included in all diagnostic offers. This is important to harmonize genetic testing. It is definitely important from the standpoint of the patients and medical practitioners who would like to see equal access and uniform services across Europe.

**STATEMENT 05: For diagnostic purpose, only genes with a known (ie,** **published and confirmed) relationship between the aberrant genotype and the pathology should be included in the analysis.**

There is a strong opinion that for genes that are responsible for a significant proportion of the defects, referred as ‘core genes', the sensitivity should not be compromised by the transition from Sanger to NGS. A strong issue is made about the *BRCA1* and *BRCA2* genes, where the sensitivity of Sanger sequencing plus deletion/duplication analysis reportedly reaches 99%. The reasoning equally applies to other genes with a high yield in diagnostics. Adding additional genes will of course increase the diagnostic yield, but this should not be at the expense of missing mutations that would previously have been detected. The incremental detection rate is thus the key determining factor in defining the core gene list and in dealing with the gaps.

**STATEMENT 06: For the sake of comparison, to avoid irresponsible testing, for the benefit of the patients, ‘core disease gene lists' should be established by the clinical and laboratory experts.**

Laboratories will apply different (technical and diagnostic) settings for NGS tests, irrespective of guidelines. Indeed, there are too many variables still that cannot be fixed through prescriptive guidelines. Therefore, we propose a simple rating system for NGS diagnostics that will warrant fair scoring and easy comparison between what different labs are offering.

1. Type A test: The lab warrants >99% reliable reference or variant calls of the coding region and flanking intronic sequences, and fills all the gaps with Sanger sequencing (or another complementary sequencing analysis), and, depending on the platform used, performs extra analysis of, for example, the homopolymer stretches.

2. Type B test: The lab describes exactly which regions are sequenced at>99% reliable reference or variant calls, and fills some of the gaps with Sanger (or other) sequencing.

3. Type C test: The type C test solely relies on the quality of NGS sequencing, while no additional Sanger (or other) sequencing is offered.

**STATEMENT 07: A simple rating system on the basis of coverage and diagnostic yield, should allow comparison of the diagnostic testing offer between laboratories.**

## Informed consent and information to the patient and clinician

The implications of a diagnostic test based on NGS depend on the procedures, platforms, filtering processes and data storage used in the laboratory. It is thus crucial that the referring physician is fully informed about the limitations and possible unfortunate effects of a genetic test.

**STATEMENT 08: The laboratory has to provide for each NGS test the following: the diseases it targets, the name of the genes tested, their reportable range, the analytical sensitivity and specificity, and, if possible, the diseases not relevant to the clinical phenotype that could be caused by mutations in the tested genes.**

The implications of a test based on NGS are mainly based on the chance of unsolicited and secondary findings. Although unsolicited findings are found in the genes linked to the tested disease, secondary findings are found in disease genes not implicated in the etiology of the tested disease.

**STATEMENT 09: The analysis pipeline of diagnostic laboratories should focus on the gene panel under investigation in order to avoid the chance of secondary findings, and be validated accordingly.**

The chance of unsolicited findings in a gene panel is very low and is mainly dependent on the genes involved. However, heterozygous mutations in recessive conditions might be detected, thereby detecting disease carriers. This will have consequences for counseling, reproductive choices, and so on.

**STATEMENT 10: Laboratories should provide information on the chance of unsolicited findings.**

Before implementing a NGS-based test, the clinical (genetic) center needs to set up an ‘unsolicited and secondary findings protocol' that has to be in accordance with the decisions of an ethical committee. It should be decided – at the laboratory, institute or national level – whether patients are offered opt-in, opt-out options to get additional information besides the initial diagnostic result. The protocol should also specify whether unsolicited findings and carrier status are reported. The laboratory has to make sure that it can manage the different options that are offered.

**STATEMENT 11: If a clinical center or a laboratory decides to offer patients an opt-in, opt-out protocol to get carrier status for unrelated diseases and secondary findings all the logistics need to be covered.**

Also, pre-test genetic counseling is necessary and should include a discussion on both expected results and the potential for unsolicited and secondary findings. Adequate information should be provided.

**STATEMENT 12: The local policy about dissemination of unsolicited and secondary findings should be clear for the patient.**

**STATEMENT 13: It is recommended to provide a written information leaflet or online available information for patients.**

## Validation

The quality of a sample is a combination of many parameters such as the amount of data produced, the proportion of PCR duplicates and the coverage. In diagnostic setting, only good-quality samples must be analyzed. It is thus essential to define the criteria to characterize high-quality targeted gene panels, exomes or genomes.

**STATEMENT 14: All NGS quality metrics used in diagnostics procedures should be accurately described.**

NGS technology requires the monitoring of run-specific and analysis/sample-specific features. Monitoring data do not have to be reported but should be used for continuous validation.

**STATEMENT 15: The diagnostic laboratory has to implement a structured database for relevant quality measures for (i) the platform, (ii) all assays, and (iii) all samples processed.**

A sample tracking method has to be used as NGS workflows are very complex and comprise multiple processing steps both in the lab and during the computational analysis.

**STATEMENT 16: Aspects of sample tracking and the installation of barcoding to identify samples, should be dealt with during the evaluation of the assay, and included in the platform validation.**

During platform validation, the laboratory has to make sure that all its devices and reagents satisfy the manufacturers' requirements. The limitations of each technology must be identified and taken into account during test development and data analysis. The laboratories may distinguish features (for validation) that belong to the platform, the specific test, or the analysis pipeline.

**STATEMENT 17: Accuracy and precision should be part of the general platform validation, and the work does not have to be repeated for individual methods or tests.**

Evidently every sequencing technology harbors its strengths and weaknesses. The bioinformatics tools must reflect these characteristics.

**STATEMENT 18: The bioinformatics pipeline must be tailored for the technical platform used.**

During pipeline validation the diagnostic specifications must be measured by assessing analytical sensitivity and specificity. For instance, algorithms that are optimized for SNP detection are less accurate for (small) insertions or deletions. The laboratory has to show that it is aware of such peculiarities and that the pipelines for variant detection are adequately tested.

**STATEMENT 19: Analytical sensitivity and analytical specificity must be established separately for each type of variant during pipeline validation.**

Any changes in chemistry, enrichment protocols, or the bioinformatics analysis platform will warrant re-validation.

**STATEMENT 20: The diagnostic laboratory has to validate all parts of the bioinformatic pipeline (public domain tools or commercial software packages) with standard data sets whenever relevant changes (new releases) are implemented.**

An in-house database containing all relevant variants provides an important tool in order to identify platform-specific artifacts, keep track of validation results, and provide an exchange proxy for locus-specific databases and meta-analyses. Typically, this database should allow for further annotations (eg, false-positives, published mutations, segregating variants, and so on), which greatly streamline the diagnostic process.

**STATEMENT 21: The diagnostic laboratory has to implement/use a structured database for all relevant variants with current annotations.**

Data storage should stick to the standard open file formats FASTQ, BAM, and VCF, which should also be used for data exchange with other laboratories. When storing the analysis results, full-log files have to be stored in addition to the analysis results. The log files should be as complete as possible, making the whole analysis from FASTQ data to the diagnostic report reproducible. Unfortunately, there is no (international) consensus yet on what should be stored. However, the storage has to be in line with national requirements and common sense.

**STATEMENT 22: The diagnostic laboratory has to take steps for long-term storage of all relevant data sets.**

Prior to launching any assay, the clinical target, that is, all coding regions plus the conserved splice sites, has to be defined. The clinical target depends on the diagnostic test and the defined gene panel.

**STATEMENT 23: The reportable range,** **that is,** **the portion of the clinical target for which reliable calls can be generated, has to be defined during the test development and should be available to the clinician (either in the report or communicated digitally).**

**STATEMENT 24: The requirements for ‘reportable range' depend on the aim of the assay.**

For instance, an exome sequencing assay with the aim to achieve a high diagnostic yield does not require additional analysis to achieve high coverage in all genomic regions covered, but needs clear communication to the clinician that the test cannot be used to exclude a particular clinical diagnosis.

The performance of the diagnostic test must be evaluated in terms of accuracy, analytical sensitivity, analytical specificity, and precision. In principle, this is not new but is generally seen as cumbersome. However, the ISO norm is very strict about this.

**STATEMENT 25: Whenever major changes are made to the test, quality parameters have to be checked, and samples have to be re-run. The laboratory should define beforehand what kind of samples and the number of cases that have to be assayed whenever the method is updated or upgraded.**

## Reporting

It is essential that NGS results are reported in a clear and consistent manner, as laboratory reports may be read by both experts and non-experts. From a practical standpoint, the clinically significant conclusions and the relevant test and test quality data should feature on the first page.

**STATEMENT 26: The report of a NGS assay should summarize the patient's identification and diagnosis, a brief description of the test, a summary of results, and the major findings on one page.**

Four examples of reports, with and without annexes, are included in the supplementary information to the guidelines.

All pathogenic (class 5) and likely pathogenic (class 4) variants have to be reported. Whether or not Unclassified Variants (UVs – class 3) are reported will depend on local practice. The latter has to be clear for the laboratory scientists, as well as for the referring clinicians.

**STATEMENT 27: A local policy, in line with international recommendations, for reporting genomic variants should be established and documented by the laboratory prior to providing analysis of this type.**

**STATEMENT 28: Data on UVs have to be collected, with the aim to eventually classify these variants definitively.**

A community activity is needed to collect and share the available information, with the aim to definitely classify the variants into pathogenic (class 5) or benign (class 1).

The policy that has been adopted by the laboratory or institute, with respect to unsolicited and secondary findings, has to be reflected in the laboratory practice and in the report.

**STATEMENT 29: Laboratories should have a clearly defined protocol for addressing unsolicited and secondary findings prior to launching the test.**

A diagnostic laboratory should not become overloaded with requests to analyze ‘old' data in the view of new findings and progress in the fields. A diagnostic request is a contract at a certain point in time. A laboratory will only be able to offer what is known, and validated, at a given point in time.

**STATEMENT 30: The laboratory is not expected to re-analyze old data systematically and report novel findings, not even when the core disease gene panel changes.**

On the other hand, if at a particular moment, it is decided – by the lab or by the community of experts in the disease – to change a variant from one class to another, the lab is responsible for reanalyzing the available data, re-issuing a report on the basis of the novel evidence, and also re-contacting referring clinicians for the patients that are possibly affected by the new status of the variant. A system effectively linking patients and variants, and allowing for the retrieval of the affected cases when variants are re-classified is necessary in such a situation.

**STATEMENT 31: To be able to manage disease variants, the laboratory has to set up a local variant database for the different diseases for which testing is offered on a clinical basis.**

## Distinction between research and diagnostics

With the increasing possibilities of genome-wide testing in diagnostics and research, the line between diagnostics and research is blurred. It is thus important to describe what can and should be done with diagnostic patient data, and for what type of analyses-specific (additional) research consent is needed. However, this does not exclude a suggestion for further research as a result of a diagnostic investigation. But the distinction between research and diagnostics has to be clear at all times.

**STATEMENT 32: A diagnostic test is any test directed toward answering a clinical question related to a medical condition of a patient.**

**STATEMENT 33: A research test is hypothesis driven and the outcome may have limited clinical relevance for a patient enrolled in the project.**

**STATEMENT 34: The results of a diagnostic test, particularly by analysis of a whole exome or genome, can be hypothesis generating.**

The use of exome or genome data obtained by NGS in a diagnostic setting are acceptable, if the aim is to obtain a genetic diagnosis and the analysis is limited to genes that are known to be linked to (the) disease.

**STATEMENT 35: Diagnostic tests that have as their primary aim to search for a diagnosis in a single patient should be performed in an accredited laboratory.**

When participating to a research project, patients and families must be aware that such a project may lead to a diagnosis or predictive information about a genetic disease. In research, clinically relevant results should only be transferred into the patient's medical record after confirmation in a diagnostic setting.

**STATEMENT 36: Research results have to be confirmed in an accredited laboratory before being transferred to the patient.**

Most laboratories set up a database of variant frequencies of all locally sequenced and/or analyzed samples (ideally healthy parents) in order to ease variant interpretation. As such a database does not contain any sensitive information, considerations based on privacy rules do not weigh against the importance of such data for improving diagnosis and healthcare.

**STATEMENT 37: The frequency of all variants detected in healthy individuals sequenced in a diagnostics and/or research setting should be shared.**

Ideally, all variants detected in disease linked genes should be submitted to databases of pathogenic variants and linked to the clinical data of the patient. The criteria and arguments used for variant classification should also be clearly described.

**STATEMENT 38: All reported variants should be shared by submission to federated, regional, national, and/or international databases.**

## Supplementary Material

Supplementary Table S5

HCMLQT negative report

Annexes of HCMLQT negative report

HCMLQT positive report

Annexes of HCMLQT positive report

Exome negative report

Exome positive report

