# Supplementary material for: Guidelines for diagnostic next-generation sequencing
Source: Eur J Hum Genet. 2015 Oct 28;24(1):2–5. doi: 10.1038/ejhg.2015.226 (PMC4795226; doi:10.1038/ejhg.2015.226)
Supplement: Supplementary Information [file ejhg2015226x1.doc]

**Supplementary information**

**Table S1**. Elements of a NGS bioinformatics pipeline

All the elements that should be part of a NGS analysis pipeline are described in table S1 as well as examples of software that can be used and output formats.

**Table S2**. QC metrics tracking for samples

Tracking QC metrics throughout the whole analysis pipeline is essential to ensure that each final report is based on diagnostics-grade read data. The most important, but by far not all, QC metrics are summarized in table S2.

**Table S3**. SNPs for sample identification

In order to make samples traceable through the whole analysis workflow, we propose to include a number of common SNPs in all panels/exome enrichments. By comparing the genotypes determined in the NGS analysis to genotypes obtained by another assay such as PCR genotyping upon sample entry, sample swaps can be easily detected. We propose to include SNPs from different chromosomes, to mitigate the risk of missing genotypes due to larger deletions or enrichment problems. The SNPs presented in table S3 are already used in some diagnostic laboratories.

**Table S4**. Quality control regions

The following exons can be used as quality control regions for comparisons and monitoring between different assays.

**Table S5**. *A priori* chance of finding a variant

*A priori* chance of finding a variant if the chance of detecting a heterozygous variant at 20X is of 99% and 0% otherwise.

**HCMLQT negative report**

Example of a one-page report of a targeted capture in which no (likely) pathogenic mutation has been found.

**Annexes of HCMLQT negative report**

Example of the annexes appended to the one-page report of a targeted capture in which no (likely) pathogenic mutation has been found.

**HCMLQT positive report**

Example of a one-page report of a targeted capture in which one (likely) pathogenic mutation has been found.

**Annexes of HCMLQT positive report**

Example of the annexes appended to the one-page report of a targeted capture in which one (likely) pathogenic mutation has been found.

**Exome negative report**

Example of a report of an exome in which no (likely) pathogenic mutation has been found. Additional information on gene panels and variant prioritization are available on request and/or on the laboratory website (http://www.genomediagnosticsnijmegen.nl/services/exome-sequencing-diagnostics).

**Exome positive report**

Example of a report of an exome in which one (likely) pathogenic mutation has been found. Additional information on gene panels and variant prioritization are available on request and/or on the laboratory website (http://www.genomediagnosticsnijmegen.nl/services/exome-sequencing-diagnostics).
