# Supplementary material for: Guidelines for diagnostic next-generation sequencing
Source: Eur J Hum Genet. 2015 Oct 28;24(1):2–5. doi: 10.1038/ejhg.2015.226 (PMC4795226; doi:10.1038/ejhg.2015.226)
Supplement: HCMLQT negative report [file ejhg2015226x7.pdf]

UZ LEUVEN

CENTRUM MENSELIJK ERFELIJKHEID
Herestraat 49, 3000 Leuven
tel: 016 34 59 03 fax: 016 34 60 60
erk.#:8-24990-92-996

Patient identification
XXXX XXXX
00000000

Gene panel (report)

Indication

HCMLQT cardiomyopathies / arrhythmias

Reason of request

The patient has a clinical diagnosis of hypertrophic cardiomyopathy (HCM).

Report

Results:

For this patient, no mutation has been found in one of the genes listed below.

Conclusion:

For this patient, no pathogenic mutation has been found. The clinical diagnosis can thus not be confirmed at the molecular level for now. This does not exclude a genetic cause of the disease in this patient or family.

Background

Cardiomyopathies are a heterogeneous group of structural heart diseases. The various forms of cardiomyopathy are classified on the basis of imaging and electrocardiographic characteristics in four diseases: hypertrophic cardiomyopathy (HCM), dilated cardiomyopathy (DCM), arrhythmogenic right ventricular cardiomyopathy (ARVC), and left ventricular non-compaction cardiomyopathy (LVNC).

Hereditary arrhythmogenic heart defects are a heterogeneous group of disorders in which abnormalities in the electrical activity of the heart can lead to arrhythmias. The different types are usually divided into the following conditions: Long QT syndrome (LQTS), Brugada syndrome (BRS), catecholaminergic polymorphic ventricular tachycardia (CPVT), short QT syndrome (SQTS), early repolarization syndrome (ERS), idiopathic ventricular fibrillation (IVF), atrial fibrillation (AF), cardiac conduction defects (CCD), and the sick sinus syndrome (SSS).

The coding exons and flanking introns of the gene panel (target capture HCMLQTv1, Nimblegen) was sequenced by massive parallel sequencing. The analysis and interpretation was done on a subpanel defined below and selected according to the clinical phenotype. If a mutation was found with this method, the result was confirmed on the basis of Sanger sequencing.

In this patient the following genes were analysed:
HCM: ACTC1, ACTN2, ANKRD1, CALR3, CASQ2, CAV3, CSRP3, GLA, JPH2, LAMP2, MYBPC3, MYH6, MYH7, MYL2, MYL3, MYLK2, MYOZ2, PLN, PRKAG2, RYR2, TCAP, TNNC1, TNNT2, TNNI3, TPM1, VCL
A deletion/duplication analysis has also been performed by MLPA (Salsa P100, MRC Holland) for the gene MYBPC3.

Three annexes complete this report (to be delivered by mail):
For the full panel of genes and a description of the technique and the bioinformatics pipeline, see Annex 1.
For quality parameters, see Annex 2 .
For an overview of the mutations and variants in this patient, see Annex 3 .
