# Supplementary material for: Guidelines for diagnostic next-generation sequencing
Source: Eur J Hum Genet. 2015 Oct 28;24(1):2–5. doi: 10.1038/ejhg.2015.226 (PMC4795226; doi:10.1038/ejhg.2015.226)
Supplement: HCMLQT positive report [file ejhg2015226x9.pdf]

CENTRUM MENSELIJK ERFELIJKHEID
Herestraat 49, 3000 Leuven
tel: 016 34 59 03 fax: 016 34 60 60
erk.#:8-24990-92-996

Patient identification
XXXX XXXX
00000000

Gene panel (report)

Indication

HCMLQT cardiomyopathies / arrhythmias

Reason of request

The patient has a clinical diagnosis of hypertrophic cardiomyopathy (HCM).

Report

Results:

This patient is heterozygous for the mutation c.98_99delCA (p.Thr33Argfs*15) in the MYBPC3 gene. The nomenclature of the mutation is based on the transcript NM_000256.3.

Conclusion:

The patient has a mutation in the gene MYBPC3. This mutation causes a frameshift that leads to an early stop codon causing the production of a truncated protein or no protein production. The mutation has not been previously described in literature, but given the nature of the mutation it has been classified as likely pathogenic. The clinical diagnosis could thus be confirmed at the molecular level. Genetic counseling is recommended. The probability that the first-degree relatives of these patients carry this mutation is 50%. Predictive testing is now possible in the family, but only after genetic counseling.

Background

Cardiomyopathies are a heterogeneous group of structural heart diseases. The various forms of cardiomyopathy are classified on the basis of imaging and electrocardiographic characteristics in four diseases: hypertrophic cardiomyopathy (HCM), dilated cardiomyopathy (DCM), arrhythmogenic right ventricular cardiomyopathy (ARVC), and left ventricular non-compaction cardiomyopathy (LVNC).

Hereditary arrhythmogenic heart defects are a heterogeneous group of disorders in which abnormalities in the electrical activity of the heart can lead to arrhythmias. The different types are usually divided into the following conditions: Long QT syndrome (LQTS), Brugada syndrome (BRS), catecholaminergic polymorphic ventricular tachycardia (CPVT), short QT syndrome (SQTS), early repolarization syndrome (ERS), idiopathic ventricular fibrillation (IVF), atrial fibrillation (AF), cardiac conduction defects (CCD), and the sick sinus syndrome (SSS).

The coding exons and flanking introns of the gene panel (target capture HCMLQTv1, Nimblegen) was sequenced by massive parallel sequencing. The analysis and interpretation was done on a subpanel defined below and selected according to the clinical phenotype. If a mutation was found with this method, the result was confirmed on the basis of Sanger sequencing.

In this patient the following genes were analysed:

HCM: ACTC1, ACTN2, ANKRD1, CALR3, CASQ2, CAV3, CSRP3, GLA, JPH2, LAMP2, MYBPC3, MYH6, MYH7, MYL2, MYL3, MYLK2, MYOZ2, PLN, PRKAG2, RYR2, TCAP, TNNC1, TNNT2, TNNI3, TPM1, VCL

A deletion/duplication analysis has also been performed by MLPA (Salsa P100, MRC Holland) for the gene MYBPC3.

Three annexes complete this report (to be delivered by mail):

For the full panel of genes and a description of the technique and the bioinformatics pipeline, see Annex 1.

For quality parameters, see Annex 2 .

For an overview of the mutations and variants in this patient, see Annex 3 .
