# Supplementary material for: Guidelines for diagnostic next-generation sequencing
Source: Eur J Hum Genet. 2015 Oct 28;24(1):2–5. doi: 10.1038/ejhg.2015.226 (PMC4795226; doi:10.1038/ejhg.2015.226)
Supplement: Annexes of HCMLQT positive report [file ejhg2015226x10.pdf]

UZ LEUVEN | CENTRUM MENSELIJKE ERFELIJKHEID

Herestraat 49, 3000 Leuven
tel: 016 34 59 03
fax: 016 34 60 60
erk.#:8-24990-92-996

## Patient identification

**Surname** XXXX
**First name** XXXX
**Gender** X
**EADnr** 00000000
**Birth date** 00/00/0000

**Sampling time** 00/00/0000 00:00
**Sample type** blood
**Sample number** XXXX
**GC code** GC000000 (A_GC000000)
**Project code** MD1-MT260-Genpanel-20

## Subpanel HCM ⊆ gene panel HCM_LQT (v1.0)

# Annex 1 General specifications of the test and analysis

The general characteristics of the full *HCM_LQT* panel are described below. The subpanel *HCM* consists of a subset of the captured genes. **Please refer to Annex 2 for the specific statisitics of the application of the subpanel *HCM* on sample *GC000000*.**

**List of the 75 genes available in gene panel *HCM_LQT*.** In total the gene panel *HCM_LQT* covers a maximum of **100%** of the combined coding regions ± 2bp intronic sequence of the following genes:

| | | | | | | |
|---|---|---|---|---|---|---|
| ABCC9 | CASQ2 | GPD1L | KCNJ5 | MYLK2 | SCN2B | TMEM43 |
| ACTC1 | CAV3 | HCN4 | KCNJ8 | MYOZ2 | SCN3B | TMPO |
| ACTN2 | CSRP3 | JPH2 | KCNQ1 | NKX2-5 | SCN4B | TNNC1 |
| AKAP9 | DES | JUP | LAMP2 | NOS1AP | SCN5A | TNNI3 |
| ANK2 | DMD | KCNA5 | LDB3 | NPPA | SGCD | TNNT2 |
| ANKRD1 | DSC2 | KCNE1 | LMNA | NUP155 | SNTA1 | TPM1 |
| CACNA1C | DSG2 | KCNE1L | MYBPC3 | PKP2 | TAZ | TRPM4 |
| CACNA1D | DSP | KCNE2 | MYH6 | PLN | TBX3 | VCL |
| CACNA2D1 | DTNA | KCNE3 | MYH7 | PRKAG2 | TBX5 | WWTR1 |
| CACNB2 | GJA5 | KCNH2 | MYL2 | RYR2 | TCAP | |
| CALR3 | GLA | KCNJ2 | MYL3 | SCN1B | TGFB3 | |

**Method** For each sample 2 $\mu$g of genomic DNA was sonicated in fragments of 300bp on average. The TruSeq DNA Sample Preparation kit of Illumina was used to make the library. After library preparation 6 samples were pooled for *in-solution* capturing with the Nimblegen SeqCap EZ kit (Lot No. xxxxxx). The enriched fragments were then amplified and sequenced on the HiSeq2500 rapid mode, 2x 100bp paired-end sequencing. This was performed in the Genomics Core facility (UZ Leuven).

**Analysis (v1.6)** After demultiplexing, the reads were aligned to the human reference genome hg19 (human_g1k_v37.fasta) using BWA (0.7.8). Duplicate reads were removed with Picard MarkDuplicates (1.118), local realignment around indels was performed with GATK RealignerTargetCreator (3.2.2), base scores were recalibrated with GATK BaseRecalibrator (3.2.2), and then variants were called with GATK HaplotypeCaller (3.2.2). Variants were annotated using RefSeq (release 65) and Cartagenia (Cartagenia Bench Lab NGS 3.1.2).

**Sanger sequencing** Mutations retained as pathogenic were confirmed by Sanger sequencing.

**General limitations of the technique** The analysis was optimised to identify base pair substitutions with a high sensitivity. The sensitivity for small insertions and deletions was lower. Deep-intronic mutations, mutations in the promoter region, repeats, large exonic deletions and duplications, and other structural variants were not detected by this test.

# Annex 2 Quality parameters

## Gene panel *HCM_LQT* applied to sample *GC000000*

- The data satisfy the proposed quality criteria.
- **99.14%** of the gene panel *HCM_LQT* is genotyped
- Total number of variants *HCM_LQT*: **1782**

## Subpanel *HCM* applied to sample *GC000000*

- **99.99%** of subpanel *HCM* is genotyped; the 26 genes transcripten of subpanel *HCM* are completely genotyped unless it is indicated otherwise (e.g. = 75%):

| | | | |
|---|---|---|---|
| ACTC1 NM_005159.4 | GLA NM_000169.2 | MYL3 NM_000258.2 | TNNC1 NM_003280.2 |
| ACTN2 NM_001103.3 | JPH2 NM_020433.4 | MYLK2 NM_033118.3 | TNNI3 NM_000363.4 |
| ANKRD1 NM_014391.2 | LAMP2 NM_002294.2 | MYOZ2 NM_016599.4 | TNNT2* NM_001001430.2 |
| CALR3 NM_145046.4 | MYBPC3* NM_000256.3 | PLN NM_002667.3 | TPM1 NM_001018005.1 |
| CASQ2 NM_001232.3 | MYH6 NM_002471.3 | PRKAG2 NM_016203.3 | VCL NM_014000.2 |
| CAV3 NM_033337.2 | MYH7 NM_000257.2 | RYR2 NM_001035.2 | |
| CSRP3 NM_003476.4 | MYL2 NM_000432.3 | TCAP NM_003673.3 | |

* (partially) genotyped by Sanger

- The table below shows the incomplete exons for some subpanel *HCM* genes that are not entirely genotyped:

| Gene | Transcript | List of incomplete exons | % gene genotyped |
|---|---|---|---|
| MYH6 | NM_002471.3 | ex29, ex37 | 99.9 |

# Annex 3 List of variants

The variants are assigned to one of the following classes: (1) benign, (2) likely benign, (3) variants of unknown significance, (4) likely pathogenic, or (5) pathogenic. Variants of class (1) and (2) are not included below.

## (Likely) pathogenic mutation(s) associated with the phenotype:

| Gene | cDNA | Genomic position | Protein | Classification | Zygosity | Heredity |
|---|---|---|---|---|---|---|
| MYBPC3 | NM_000256.3:c.98_99delCA | Chr11:g.47372983_47372984delTG | p.Thr33Argfs*15 | likely pathogenic | heterozygote | AD |

## There is no variant of unkown significance that could be associated with the phenotype.
