# Supplementary material for: Guidelines for diagnostic next-generation sequencing
Source: Eur J Hum Genet. 2015 Oct 28;24(1):2–5. doi: 10.1038/ejhg.2015.226 (PMC4795226; doi:10.1038/ejhg.2015.226)
Supplement: Exome positive report [file ejhg2015226x12.pdf]

EXOME SEQUENCING REPORT

Clinical geneticist

Genome Diagnostics Nijmegen
Department of Human Genetics
Radboud University Medical Center

848 Genome Diagnostics
PO box 9101
6500 HB Nijmegen, the Netherlands

Tel: +31 24 3613799
Fax: +31 24 3616658
Email: gen@umcn.nl
www.umcn.nl/genomediagnostics
www.genomediagnosticsnijmegen.nl

Our reference: X00-00000 / 00-00000
Your reference: 0000000000

Nijmegen, 00-00-0000

PERSONAL DETAILS

Name: X. XXXX
Date of Birth: 00-00-0000
Sex: F

Date of request: 00-00-0000
Indication: muscle disease (gene panel analysis, NGS)
Reason for referral: hypotonia, joint contractures and joint laxity

SAMPLE DETAILS

Material	Date of receipt	DNA-number
DNA	00-00-0000	DNA00-00000

RESULTS AND MOLECULAR INTERPRETATION

Using exome sequencing, a pathogenic mutation was detected in one of the genes associated with muscle disorders (gene panel version DGD_14112014).

COL6A3; Chr2(GRCh37):g.238269763C>T; NM_004369.3:c.6210+1G>A; r.spl?; heterozygous; de novo. The nomenclature used follows the HGVS mutation nomenclature guidelines (www.hgvs.org).

Mutations in COL6A3 cause either Bethlem or Ullrich congenital myopathy (OMIM#254090 and #158810), which both may be inherited in a dominant or recessive way. The donor splice site mutation in the counselee was not detected in either parent, suggesting a de novo event. The effect of the mutation is predicted to result in in-frame skipping of exon 16, which is consistent with a dominant-negative effect of this mutant.

CONCLUSION

The mutation in the COL6A3 gene is very likely the cause of the counselee’s hypotonia, joint contractures and joint laxity.

TEST DESCRIPTION

Exome enrichment (Agilent SureSelectXT Human all Exon 50 Mb), exome sequencing (Illumina HiSeq2000TM), read alignment (BWA) and variant calling (GATK) were done at BGI-Europe (Denmark). Variant annotation, selection, and prioritizing for pathogenicity was done by the department of Human genetics, Radboudumc, using an in-house developed strategy. This pipeline also filters the data for the genes defined in the gene panel, if applicable (information about the gene panel and its prioritization is available on request, and accessible from our website). Confirmation of reported variants by an independent technique (such as Sanger sequencing) has only been performed for low-quality variants (GATK quality scores). This has been validated to have a >99.9% reliability of the non-confirmed variants to be present and to be ‘de novo’ (if applicable).

DISCLAIMER

This exome sequencing test produces data that cover the majority of the exome. Nevertheless, some areas of the exome are poorly covered or absent from the data. Thus, mutations in those regions may remain unidentified. Furthermore, some types of mutations (such as repeat expansions, copy-number variants, mitochondrial-DNA mutations and areas beyond the exome, including introns and promoters) will not have been detected by this test. The gene panels are updated regularly, but may be incomplete due to the continuous identification of novel genes in human disease. Specific information about the coverage, gene panels, etc. is available on request. The aim of this test is to reveal the cause of the disorder in the counselee, not to reveal the putative carrier status of recessive disorders. Carrier status of a given disorder may be requested in particular situations, ie in case of consanguinity between the counselee and his/her partner, or if the partner of the counselee has a molecularly-confirmed disorder. Due to small likelihood (<1:1000) of a false-positive report (see test description), there is a small risk of a withdrawal of a previous conclusion. This should be considered during counseling of the proband, or family members (cascade screening). Especially in future prenatal testing, a prior request of confirmation of a mutation is indicated.

With kind regards,

Y. YYYY, PhD	Z.ZZZZ, PhD
Clinical Laboratory Geneticist	Clinical Laboratory Geneticist *

This report has been signed and authorised electronically (*).
